# Embryo selection with artificial intelligence: how to evaluate and compare methods?

**DOI:** 10.1007/s10815-021-02254-6

**Published:** 2021-06-26

**Authors:** Mikkel Fly Kragh, Henrik Karstoft

**Affiliations:** 1 Department of Electrical and Computer Engineering, Aarhus University, Aarhus N, Denmark; 2 Vitrolife A/S, Viby J, Denmark

**Keywords:** Embryo selection, Artificial intelligence, Model evaluation and comparison, Selection bias

## Abstract

Embryo selection within in vitro fertilization (IVF) is the process of evaluating qualities of fertilized oocytes (embryos) and selecting the best embryo(s) available within a patient cohort for subsequent transfer or cryopreservation. In recent years, artificial intelligence (AI) has been used extensively to improve and automate the embryo ranking and selection procedure by extracting relevant information from embryo microscopy images. The AI models are evaluated based on their ability to identify the embryo(s) with the highest chance(s) of achieving a successful pregnancy. Whether such evaluations should be based on ranking performance or pregnancy prediction, however, seems to divide studies. As such, a variety of performance metrics are reported, and comparisons between studies are often made on different outcomes and data foundations. Moreover, superiority of AI methods over manual human evaluation is often claimed based on retrospective data, without any mentions of potential bias. In this paper, we provide a technical view on some of the major topics that divide how current AI models are trained, evaluated and compared. We explain and discuss the most common evaluation metrics and relate them to the two separate evaluation objectives, ranking and prediction. We also discuss when and how to compare AI models across studies and explain in detail how a selection bias is inevitable when comparing AI models against current embryo selection practice in retrospective cohort studies.

## Introduction

During the past few years, artificial intelligence (AI) has heavily influenced innovation and research within the field of in vitro fertilization (IVF). In the near future, AI applications may assist or even fully automate IVF procedures such as assessing gamete quality, selecting sperm during intracytoplasmic sperm injection (ICSI), collecting oocytes, assisting with patient stimulation protocols, donor matching, or selecting and ranking embryos for transfer and cryopreservation [1]. Furthermore, AI may help optimize and standardize clinical processes by introducing predictive maintenance in IVF instruments and automatically extract and analyze key performance indicators in order to carry out continuous quality control [2].

Several studies have reviewed AI algorithms and their uses for various applications within IVF [2–8]. In this paper, however, we focus specifically on embryo evaluation and selection, as this is currently the most active research area of applying AI within IVF, with more than 10 papers published in 2020. Automated embryo evaluation using machine learning or computer vision based on embryo images has been an active field of research for more than a decade [9, 10]. Yet, within the past few years, many of the publications have focused more on commercialization and competition rather than methodological novelties and technical details of the AI [11–15]. Instead, they seem to focus on reporting large datasets, high performance values based upon a variety of metrics, and ability to surpass human/embryologist performance. The evaluation methods and clinical endpoints vary considerably across studies, and performance comparisons are sometimes made on completely different data foundations (patient demographics, unbalanced data, sub-cohorts, etc.). It has therefore become evident that the research community does not agree on a standard for how to report and compare performances of AI models. A recent review by [8] supports this claim, underlining “the importance of transparency and standardization in reporting AI models”. The review also points clinicians and researchers towards two established and internationally accepted guidelines for how to report clinical prediction models (TRIPOD: [16]) and how to assess potential risk of bias in models or model comparisons (PROBLAST: [17]). Although the guidelines primarily address regression models, most items still apply to machine learning and AI methods as well. Additionally, a new guideline (TRIPOD-AI) is currently being developed specifically targeting reporting of AI models [18].

Traditionally, the main objective of embryo evaluation has been to rank embryos within a patient cohort according to their potential to implant. In this context, the actual predictions by the AI model (e.g., between 0 and 1) for each embryo is of limited relevance, as long as the order (ranking) of the values within the cohort correlates with the likelihood of implantation. In clinical practice, a pure ranking model can help sort the embryos within a cohort, but may not be useful for deciding which of them (if any) are viable enough for transfer or cryopreservation. Recent approaches, however, attempt to provide predictions that directly represent the likelihood of implantation, thus adding a second objective to embryo evaluation in the form of probability estimation. A prognostic estimate of implantation probability for each embryo, possibly incorporating patient characteristics, can thus help in the decision process regarding which embryos to prioritize for transfer and which to cryopreserve. As such, prognostic prediction may also simplify and improve communication to patients. In this paper, we distinguish between the two objectives and categorize them as **ranking** and **prediction**, respectively. The two objectives relate directly to model discrimination and model calibration [19] that each have separate performance measures. Therefore, when evaluating AI models for embryo evaluation, one needs to be aware of which objectives (ranking vs. prediction) that have been optimized and evaluated for. Similarly, it is important to notice how the evaluation reflects the intended use of a model. For instance, a model evaluated solely on transferred embryos, implicitly assumes manual preselection by embryologists of embryo sub-cohorts and is thus intended to be used as a supplement to manual, human evaluation. A fully automated model with intended use to analyze all embryos within a cohort, on the other hand, needs to be evaluated across all embryos [2].

The review by [8] provides a list of criteria, which researchers and clinicians can use to evaluate studies about AI models. The list includes assessments of model generalization, dataset balance requirements, bias considerations, and guidelines for the best performance metrics. Although the review successfully points out some of the important challenges and pitfalls concerning training and evaluation of AI models, it provides a too simplified view of some of the topics. In this paper, we therefore elaborate more technically on four of the major topics that seem to divide how current AI models are trained, evaluated and compared:

- In “Data foundation,” we address the data foundation, on which a study is based. Here, we provide a scheme to categorize AI models based on their embryo population and outcome and use this to illustrate why model comparisons are often unjustified. We also explain why we cannot simply define universal requirements for balancing datasets or for splitting datasets into training, validation and test sets.
- In “?? ??” we present the most common evaluation metrics, discussing pros and cons while relating them to their dependency on data balancing and to the two objectives, ranking and prediction.
- In “Sample size,” we illustrate how sample size of the test set affects the certainty of the most common performance measures.
- In “Bias in model comparisons,” we demonstrate and discuss bias in model comparisons. Here, we provide an in-depth explanation based on simulated data of how selection bias is inevitable when comparing AI models against current embryo selection practice in retrospective cohort studies.
- Finally, in “Discussion,” we summarize our main points and suggestions and discuss how future studies including AI methods can strengthen their evaluations and reduce bias in reported metrics and model comparisons.

Throughout the sections, Table 1 is used to exemplify disparities between data foundations and evaluation methods for different embryo evaluation studies using AI. The table lists 13 studies that all used pregnancy-related outcome to train and evaluate AI models on image data. The studies are categorized in terms of data input, evaluated outcome, embryo population, inclusion of human vs. AI comparison, and metrics used for the evaluation.

**Fig. 1:**
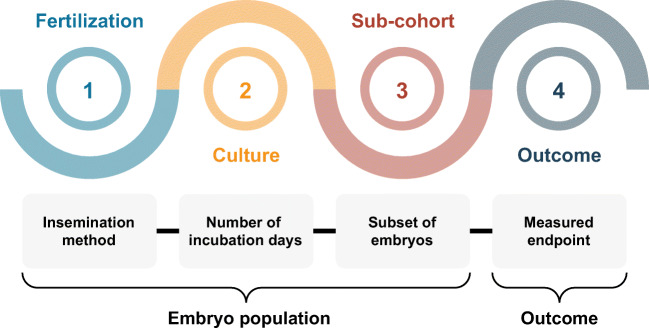
Example scheme for reporting embryo population and outcome. A study reporting prediction of live birth on transferred day 5 blastocysts fertilized by ICSI would have the embryo population *ICSI-D5-Blastocyst-Transfer* and outcome *live birth*

**Table 1:** List of studies that used AI on image data to predict or rank embryos based on pregnancy outcome. The reported information only concerns evaluation of pregnancy-related outcomes. Therefore, if a study includes additional tasks such as blastocyst prediction, these are not included in the table

Reference	Input	Outcome	Embryo population^a^	Human vs. AI^b^	Metrics^c^
[20]	Static image	Fetal heartbeat	*-D5-blastocyst	✓	Accuracy, AUC
[21]	Static image, patient age	Beta-HCG	*-D5/D6-blastocyst	-	Accuracy, sensitivity, specificity, PPV, NPV, FPR, FNR, F1, AUC
[13]	Static image, patient age, blastocyst age, lab settings	Ploidy/beta-HCG	*-D5/D6-blastocyst	✓	Accuracy, sensitivity, specificity, PPV, AUC, NDCG
[22]	Time-lapse video	Fetal heartbeat	ICSI-D3-*, ICSI-D5-*	$\left (\checkmark \right )$	Sensitivity, PPV, AUC
[11]	Time-lapse video	Fetal heartbeat^2^	*-D5-*	-	AUC
[12]	Static image	Fetal heartbeat	*-D5-blastocyst	✓	Accuracy, sensitivity, specificity
[23]	Static image	“pregnancy”	*-*-blastocyst	-	Accuracy, sensitivity, specificity
[24]	Static image	Live birth	*-D5/D6-blastocyst	$\left (\checkmark \right )$	Accuracy, sensitivity, specificity, AUC
[25]	Static image	Live birth	*-D5-blastocyst^1^	-	Accuracy, sensitivity, specificity, PPV, NPV, AUC
[26]	Static image, annotations, patient info (age, BMI, ...)	Live birth	*-D5/D6-blastocyst	$\left (\checkmark \right )$	Accuracy, sensitivity, specificity, informedness, AUC
[27]	Static image, annotations, patient info (age, BMI, ...)	Live birth	*-D5/D6-blastocyst	-	Accuracy, sensitivity, specificity, informedness, PPV, NPV, AUC
[14]	Time-lapse video	Fetal heartbeat	*-*-*	✓	PPV, NPV, AUC
[15]	Time-lapse video	Fetal heartbeat	*-D5/D6-*	$\left (\checkmark \right )$	AUC

^a^ The notation for *Embryo population* is explained in “Data foundation” and visualized in Fig. 1

^b^ Human vs. AI comparisons are discussed in “Bias in model comparisons”

^c^ All metrics are explained in detail in “Evaluation metrics: which performance measure to use?”

^1^ Only aneuploid miscarriages (confirmed with genetic testing of chorionic villus samples) were included as negative live births

^2^ Negative fetal heartbeat was assumed for all non-transferred embryos that had “failed or abnormal fertilization, grossly abnormal morphology or aneuploidy from preimplantation genetic testing”

## Data foundation

An important parameter that often differs between AI models is the data foundation used to train and evaluate them. Some models seek to automate the embryo selection process completely, assuming no preselection of embryos by embryologists [11, 15]. Such models need to evaluate the performance not only on transferred embryos, but also on low quality embryos at different developmental stages, in order to ensure that the evaluation data is representative of prospective use [2]. Other models seek to differentiate between previously transferred embryos [12, 14, 20–22, 24]. When only evaluating on transferred embryos, such models assume that an embryologist first preselects potentially transferable embryos (e.g., day 5 blastocysts). Thus, they are developed on datasets consisting of more similar embryos in terms of incubation time, developmental stage and quality.

In order to compare AI models, it is essential to note the embryo population and which outcome was used for training and (more importantly) evaluation. In this paper, we use a population-outcome scheme to characterize AI models used for embryo evaluation by their data foundation. Figure 1 illustrates the scheme consisting of four different attributes:

- **Fertilization**: which method(s) of fertilization were included? IVF, ICSI or both?
- **Culture**: for how long were the embryos incubated? (e.g., 5 days)
- **Sub-cohort**: which of the available embryos were included? (e.g., blastocysts, hatched blastocysts, euploid, fresh, cryopreserved)
- **Outcome**: what was the measured endpoint? (e.g., fetal heartbeat, live birth)

The embryo population is characterized by the first three attributes and provides a description of which embryos were included in a study, whereas the outcome defines the clinical endpoint (or ground truth) that the model was evaluated against. For instance, a study reporting prediction of live birth on all embryos transferred on day 5 or 6 after fertilization by ICSI would have the embryo population ICSI-D5/D6-* and outcome *live birth*. By considering *live birth* as outcome, only transferred embryos were considered. D5/D6 means that the all embryos were incubated for either 5 or 6 days, whereas * denotes that all transferred embryos within a cohort were included (not only fresh transfers or euploid embryos for instance). Another example could be a study reporting prediction of ploidy on all day 5 blastocysts that had undergone PGT, but not necessarily transfer. This would be characterized by the embryo population *-D5-Blastocyst and outcome *euploid*.

When applying the rather simple scheme in Fig. 1 onto the studies in Table 1, it is clear that both embryo population and outcome vary considerably between studies. In addition, attributes such as patient demographics, egg donation, culture media, image quality criteria, and other clinical settings may also be relevant in characterizing a specific population of embryos. For instance, if a study applies data cleaning based on image quality [13, 20], it effectively changes the embryo population on which the model is intended to be used clinically and upon which it can be compared to other models. If not addressed explicitly in terms of intended use, such exclusion of outliers, occluded embryos, or missing data may introduce a risk of bias [17].

Another factor that can greatly influence the reported performance is the characteristics of the input provided to the model. Similar to embryo population and outcome, Table 1 shows considerable variation in the provided inputs. Some models include a single static image of the embryo, whereas others include a time-lapse video of the developing embryo. Other models may include parameters such as patient age, previous number of attempts, stimulation protocol, clinic-specific settings, and manual annotations of morphokinetic and morphological parameters. Adding such inputs may improve performance metrics drastically. For instance, implantation models will generally improve their overall discrimination performances when including age (patient and/or oocyte) as an input variable. For predicting pregnancy probabilities, this may improve a model considerably, as it can adapt to the general decrease in success rates with age. For embryo ranking, however, increased discrimination performance across all age groups may not actually improve ranking ability within individual patient cohorts. That is, when applying the model in an actual clinical setting to discriminate embryos within a single patient cohort (where the patient age is a constant), the ranking potential may be unchanged [28].

The large variation across both input, embryo population and outcome makes comparison of AI performance results across studies difficult if not impossible. For instance, Fernandez et al. [4] compare accuracy measures across different studies and datasets, without taking into account the different embryo populations and distributions of labels in the test sets. Miyagi et al. [26] conclude that their predictive results are good, because their area under the curve (AUC) performance values on patient ages ≥ 38 years are higher compared to AUC values obtained across all age groups in a different study. Such comparisons are invalid, simply because the embryo populations in the different studies are different. Similarly, a considerable bias can appear if compared models measure different outcomes [29]. Paired analyses, or direct comparisons, evaluating different models on the exact same dataset thus seem to be the only appropriate and valid comparison available [16]. And even then, pitfalls still exist when evaluating an embryo population upon which one of the compared models was involved in the decision process, e.g., by deciding which embryos to transfer. “?? ??” elaborates on this potential selection bias when comparing prediction models in retrospective cohort studies.

### Data split

To develop an AI model, a representative and hopefully diverse dataset first has to be collected. The dataset is typically divided into development (training and validation) and test subsets. In this context, it is important to split the dataset on patient or treatment level, such that embryos from the same couple are not divided into different subsets. Splitting simply on embryo level could introduce a bias due to correlation between both the embryo images/videos and the associated outcomes. For constructing the test set, an even stronger approach is to split the dataset by time, such that the model is trained on an early time period and evaluated on a later time period [16].

Because AI models often contain millions of parameters, they easily “overfit,” meaning that they memorize the training examples directly instead of learning to generalize to new examples based on similar features. The development dataset is therefore normally split into a training and validation subset. Preferably, this is done with cross-validation in order to ensure generalization and mitigate bias. Cross-validation may even be stratified such that examples from each relevant subgroup are evenly distributed across the different folds. The validation set is used to continuously monitor the generalization power of the AI model on unseen data. Often, the generalization power will increase during the first part of training. After a while, it might start to decrease, indicating that the model is overfitting. Since the validation data are typically used to tune a number of “hyperparameters” such as deciding the optimum type and size of the AI model, or the ideal time duration to train the model, the performance on the validation set during a hyperparameter search gradually becomes slightly biased. Therefore, a completely separate and independent test set must be used as a final step for evaluating and reporting an unbiased estimate of the generalization performance of the developed model. For clinical prediction models, this step is often referred to as interval validation, because the evaluation is applied on a data subset representing the same population and distribution as the training set of the model. As this will generally provide an optimistic estimate of generalization performance, subgroup analyses (or stratified performance evaluations) can be conducted in order to reveal problems caused by a potential mismatch between training and deployment domains [30]. A stronger evaluation procedure referred to as external validation, on the other hand, tests the performance of the model in a new setting, such as a new clinic, new time period, new country, or even a new population that was not included during model development [16].

Traditionally, a common ratio for splitting a dataset into training, validation and test subsets is 70%/15%/15%, such that 15% of the entire dataset is held out for testing [31]. However, in practice, it is impossible to define a one-size-fits-all split strategy, as overfitting is greatly affected by technical training characteristics such as number of model parameters, regularization methods, potential pretraining on other datasets (transfer learning) such as ImageNet [32], or use of unsupervised or semisupervised learning. Therefore, one model might require only a limited (labeled) training set to, e.g., finetune a subset of model parameters, whereas another model might need a very large training set in order to fit all parameters from scratch. For instance, the data splitting used for the ImageNet challenge is 88%/4%/8% [32], thus allocating 1.2 million images for training, but still a notable amount of 100,000 images for testing. For this reason, less emphasis should be put on split percentages and size of the training set. Instead, the absolute size of the test set determines what claims of performance can be made, and with what statistical certainty potential superiority over humans or other models can be claimed. In “Sample size,” the influence of sample size on performance certainty is demonstrated for different evaluation metrics.

In addition to reporting prediction results on an independent test set (internal validation), some multicentric studies specifically address generalization performance across clinical practices by carrying out cross-validation in the form of clinical hold-out tests [11, 15]. This can be seen as an intermediate step between internal and external validation, as the performance is evaluated on new clinics, however not by the final prediction model, but instead separate development models expected to resemble the final model. Similar cross-validation analyses could split the dataset across IVF/ICSI fertilization, age groups, ethnicities, years of treatment, etc. to reveal potential model or dataset biases such as “spectrum bias” and “historical bias” [7]. However, a pure external validation requires the new setting (e.g., clinic) to be held out entirely during model development such as the so-called “double-blind test sets” reported by [12].

### Unbalanced data

A fundamental problem in machine learning is how to deal with unbalanced datasets, also known as class imbalance. In traditional IVF, the number of negative outcomes often exceeds the number of positive outcomes, as the overall success rate of transferred embryos is typically only around 30%. Therefore, a dataset of transferred embryos will often contain around two times as many negative outcomes as positives.

The issue concerning class imbalance can be split into two separate problems: (1) training a model, and (2) evaluating a model. For training an embryo evaluation model, class imbalance has been pointed out as a major challenge [6, 8], as the model will incorporate a prior probability (bias) related to the success rate within the training set. A simplistic way to handle this is to balance the dataset by excluding a large amount of negative outcomes, such that the model is trained on an even amount of positive and negative outcomes. More sophisticated ways that avoid exclusion of data examples include oversampling positive outcomes or adjusting the optimization algorithm by weighting the objective function used during training such that the cost of misclassifying positive and negatives examples is effectively equal.

However, class imbalance is not necessarily unwanted. If the goal of the model is to estimate the actual probability of pregnancy, and the model is to be applied in an embryo population similar to the one represented by the dataset, the model should in fact learn a prior probability (bias) of pregnancy. In this case, neither the training set nor the test set used for evaluating the model should be balanced. Instead, they should both represent the (realistic) distribution of embryos, on which the model is intended to be used.

Two recent review articles on clinical prediction models in IVF argue both for and against data balancing. Curchoe et al. [8] argue that “any validation testing should be performed with a balanced data set”. However, Curchoe et al. [2] argue that AI models need to trained and validated on datasets that are representative of the data they will be used on prospectively, which essentially is unbalanced.

One compromise could be to report metrics that are independent of class imbalance, such as sensitivity, specificity, and area under the receiver operating characteristic curve (explained in the following section). Additionally, test set prevalence (proportion of samples that are positive) should always be reported, such that it is possible to assess class imbalance and compare all metrics to random chance or naive guessing. For instance, for a dataset with a prevalence of 30%, naive guessing by always predicting “negative” results in a naive accuracy of 70%. Model performance should then be compared to a random chance of 70% instead of the usual 50%.

## Evaluation metrics: which performance measure to use?

As outlined in Table 1, a variety of different metrics are used to evaluate the performance of AI models for embryo evaluation. Some of these are binary classification metrics, calculated based on a confusion matrix that links actual observations (pregnancy or not) with predicted (binary) values. While most prediction models are continuous in nature, binary values often arise from dichotomization, that is, introducing a prediction threshold resulting in binary predictions. However, dichotomization may remove relevant information and furthermore assumes a single clinically relevant threshold [16], which can potentially cause substantial bias in estimated classification measures when the threshold is chosen to maximize performance [17]. Therefore, another group of metrics related to discrimination operate on continuous prediction values. The area under the curve (AUC) belongs to this group as it aggregates performance across all possible prediction thresholds. Each metric has its advantages and disadvantages, some being easily interpretable but biased by prevalence, while others being unbiased but clinically less relevant.

### Binary classification metrics

**Fig. 2:**
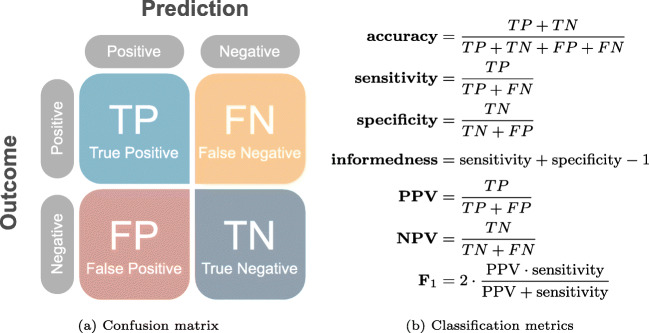
Confusion matrix and definitions of common binary classification metrics

Figure 2 illustrates a confusion matrix along with formulas for calculating accuracy, sensitivity, specificity, positive predictive value (PPV), negative predictive value (NPV) and F_1_-score.

**Accuracy** (proportion of correct predictions) is probably the most intuitive and widely used performance measure. However, it is also misleading if the evaluated dataset is unbalanced (e.g., more negative than positive pregnancies). For instance, Chen et al. [33] used a highly unbalanced dataset for inner cell mass grading and reported an accuracy of 91%, which seems high compared to a random-guessing accuracy of 33%. However, due to the dataset imbalance, merely predicting the class that occurred most frequently would have resulted in an accuracy of 83%. Therefore, reported accuracies should always be compared to a baseline corresponding to naive classification performance. In the case of pregnancy prediction, this baseline depends on the prevalence of the measured outcome.

**Sensitivity** and **specificity** describe the proportion of pregnancies that were predicted correctly as positive and the proportion of failed implantations that were predicted correctly as negative. Contrary to accuracy, these metrics are both independent of prevalence. As such, they are sometimes summarized by the single metric **informedness** (or Youden’s J statistic [34]) used to replace the misleading accuracy metric.

**Positive predictive value (PPV)** (also called precision) and **negative predictive value (NPV)** describe the proportion of positive predictions that were in fact pregnancies and the proportion of negative predictions that were in fact failed implantations or miscarriages. PPV and NPV are often considered as clinically relevant metrics, since they describe the probabilities of pregnancy/no-pregnancy given a positive/negative prediction. However, as both metrics depend on prevalence, PPV and NPV alone do not describe if the predictions are better than random.

**F**_**1**_**-score** is yet another summarized metric, although rarely used for evaluating embryo evaluation models. It is the average (harmonic mean) of PPV (precision) and sensitivity (recall) and is often used in information retrieval for testing prediction performance on positive predictions and outcomes only, thus ignoring true negatives (TN). For instance, a face detection model should be evaluated on how many actual faces it detects in an image and how many false detections it introduces in the image. It should not, however, be evaluated on the true negatives (TN), being the (infinitely many) positions in the image correctly identified as not containing faces. For embryo evaluation, however, true negatives represent non-viable embryos that should be taken into account in order to minimize time to pregnancy and associated costs. Therefore, F_1_-score does not provide a full picture and should, in our opinion, only be used to provide relative model comparisons with paired analyses on the same test set.

### Model-wide metrics

**Fig. 3:**
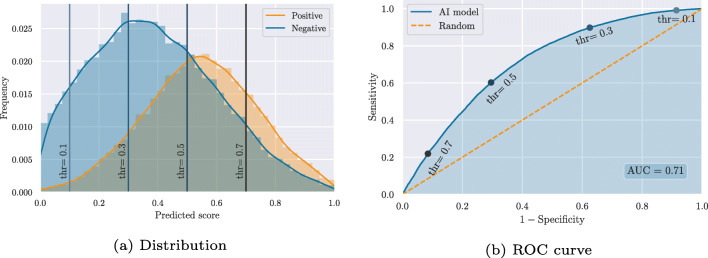
Example of a hypothetical distribution of predicted scores across positive and negative implantation outcomes and the corresponding receiver operating characteristic (ROC) curve

All of the above metrics operate on binary predictions. Most AI models, however, provide continuous output values that need to be thresholded before providing a binary prediction. That is, a prediction of which embryos are considered positive and which are considered negative by the model. While a threshold of 0.5 is often assumed for models that predict values between 0 and 1, the optimum threshold may be either smaller or larger and may not generalize between different patients and clinical practices. Moreover, for a specific threshold to be useful in a clinical setting, the continuous predictions should first be calibrated against observed outcomes [17]. That is, the predicted value should correspond to the probability of, e.g., implantation [19]. In such cases, a (calibrated) threshold may make sense in determining which embryos are useful for transfer or cryopreservation and which embryos should not be used. The primary objective of current embryo evaluation models, however, is to rank embryos within a single patient cohort according to their implantation potential and thus the order in which they should be transferred to minimize time to live birth. In this context, the AI model should not be evaluated on binary predictions, but instead on its ability to rank the embryos, such that the predicted values correlate with implantation potentials. The area under the curve (**AUC**) of the receiver operating characteristic (**ROC**) constitutes such a ranking metric, which is independent of a specific threshold. This is sometimes referred to as a model-wide metric, because it summarizes the performance across the entire model or score range. The AUC is calculated by first constructing a ROC curve, mapping the relationship between sensitivity and specificity for all possible thresholds. Figure 3 illustrates a hypothetical distribution of predicted scores across positive and negative implantation outcomes. By applying a threshold on the predicted scores, a confusion matrix like the one in Fig. 2a can be constructed and used to calculate a sensitivity and specificity. This corresponds to a single point on the ROC curve. For instance, Fig. 3 illustrates how four different thresholds on the predicted score distribution relate to points on the ROC curve. The example also illustrates how *thr = 0.5*, in this case, is not the optimum threshold if we want to optimize for either accuracy, informedness or F_1_-score. In fact, the maximum accuracy is achieved at a score threshold of 0.54, informedness at 0.47, and F_1_-score at 0.40.

The AUC (or concordance (c) statistic) is simply defined as the area under the ROC curve. As such, it provides a combined measure of discrimination ability across all possible thresholds. Since it is calculated based on sensitivities and specificities that are both independent of prevalence, AUC is also independent of prevalence. The AUC can be interpreted as the probability that a randomly chosen positive sample (pregnancy) is ranked higher than a randomly chosen negative sample (failed implantation). Since it is calculated across the entire population of embryos from various patients and clinics, AUC does not directly reflect ranking performance within single patient cohorts. Unfortunately, cohort-specific evaluations are typically unattainable, as outcome information is only available for transferred embryos that usually constitute a small fraction of the embryos in a cohort. However, various approaches that deal with clustered data may be used to report, e.g., average AUCs across different clinics in order to provide discrimination performances that are not biased by, e.g., clinical differences [16, 35].

AUC has been falsely accused of being influenced by class imbalance [12, 21] with the conclusion that “the metric cannot be trusted in highly unbalanced data” [8]. However, as described above, AUC is independent of prevalence and thus not influenced by unbalanced datasets. That is, if a dataset includes, e.g., 10 times more negative than positive outcomes, the AUC remains the same theoretically as if they were balanced, as long as the score distributions of positive and negative samples are unchanged. In Fig. 3a, this means that the difference in height between the distributions of positive and negative samples does not influence AUC. The above studies may have confused class imbalance with unequal misclassification cost, that is, the case in which false positives (FP) and false negatives (FN) are weighted unequally. In IVF, a false positive translates to a failed implantation or miscarriage after transfer of a chosen embryo, whereas a false negative translates to a missed pregnancy because the embryo was incorrectly deprioritized for transfer. Therefore, both misclassification types have costs, and the optimal compromise between these may differ between clinics and patients, as the trade-off is defined by multiple factors including time to pregnancy, financial costs, and emotional costs. Therefore, we suggest that AUC values should generally be accompanied by ROC curves that provide a full picture of performances across different thresholds and allow the reader to lookup sensitivity and specificity at their preferred trade-off (threshold). PPV and NPV can then be derived by weighting the sensitivity and specificity by prevalence:

1 $$ \begin{array}{@{}rcl@{}} PPV &=& \frac{\text{sensitivity} \times \text{prevalence}}{\text{sensitivity} \times \text{prevalence} + \left( 1-\text{specificity}\right) \times \left( 1-\text{prevalence}\right)} \end{array} $$

2 $$ \begin{array}{@{}rcl@{}} NPV &=& \frac{\text{specificity} \times \left( 1-\text{prevalence}\right)}{\left( 1-\text{sensitivity}\right) \times \text{prevalence} + \text{specificity} \times \left( 1-\text{prevalence}\right)} \end{array} $$

Although not reported in any of the studies in Table 1, an alternative representation to the ROC curve is a precision-recall (**PR**) curve. The curve maps the relationship between PPV (precision) and sensitivity (recall) and is therefore independent of true negatives. Similar to the ROC curve, it can be summarized using an area under the curve and is then specifically termed **PR AUC** to avoid confusion between the two. PR AUC has the same benefits and disadvantages as the F_1_-score mentioned above, however with the important difference of providing a performance measure across all possible thresholds.

Another ranking metric reported by [13] is the normalized discounted cumulative gain (**nDCG**) [36]. It measures the ranking quality within a cohort by weighting embryos by their relevance and their position in the sorted list of model scores. In the study by [13], relevance was measured in terms of euploid/aneuploid outcomes of preimplantation genetic testing (PGT). nDCG provides a ranking measure between 0 and 1, with 1 indicating perfect ranking. That is, nDCG = 1 when all euploid embryos within a cohort have higher scores than all aneuploid embryos. The metric is highly relevant for evaluating the ability of a model to rank embryos according to PGT outcomes, whereas it may be less relevant for evaluating ranking ability on transferred embryos according to pregnancy outcome. This is because PGT results are obtained in parallel on multiple embryos from a cohort irrespective of the ranking, whereas pregnancy outcomes are obtained sequentially according to the ranking until the first positive pregnancy occurs. Effectively, this limits the data foundation on which a meaningful analysis for transferred embryos can be carried out unless all embryos are cryopreserved and eventually transferred with a known outcome.

### Clinical usefulness

All of the above mentioned metrics address model discrimination, that is, the ability of a model to discriminate between examples with positive and negative outcomes. However, the ability of a model to discriminate on all embryos, transferred or not, from a single or even multiple clinics does not necessarily relate to how useful the model would be in clinical practice. To address this question, model calibration curves can be used to report clinical agreement between model predictions and observed outcomes [37, 38]. This relates to the prediction objective of embryo evaluation, rather than ranking.

After training an AI model using, e.g., a neural network, the predicted scores of the model typically lie between 0 and 1, with 1 indicating a higher likelihood of pregnancy than 0. However, this does not mean that the predicted scores directly represent pregnancy probabilities. Therefore, before evaluating how the scores relate to observed success rates, AI models typically need to be calibrated. A prerequisite to successful calibration, however, is that a monotonic relationship exists between predicted scores and success rates. This can be measured using the Spearman’s rank correlation coefficient *ρ* which is 1 in case of a perfect monotonic increasing relationship and close to 0 in case of a weak relationship. [22] has used this to compare the relationship between model scores and observed success rates, grouped by deciles of scores. If *ρ* is close to 1, model predictions can be calibrated using, e.g., a logistic regression model in order to obtain a linear relationship between scores and success rates. Here, either the training set or the validation set can be used to fit calibration coefficients. However, whereas data balancing may have helped during training of the AI model, it is essential that calibration coefficients are estimated without artificial data balancing. Otherwise, the predicted probabilities by the model will be biased [17]. To limit overfitting, shrinkage techniques may be used [16, 19], either within the AI model itself or in the calibration method.

**Fig. 4:**
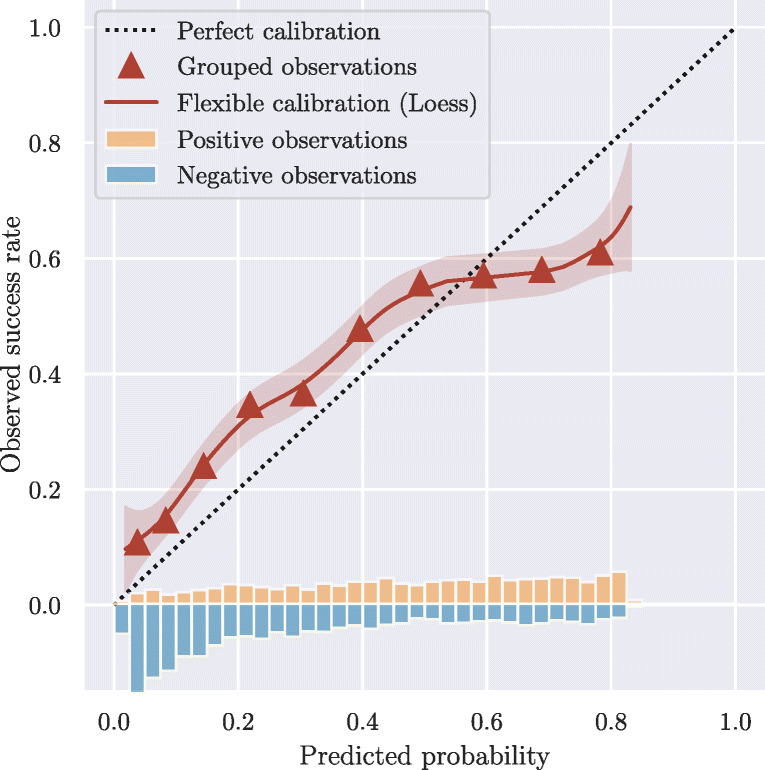
Calibration plot linking predicted probabilities to actual success rates. Grouped observations (triangles) represent success rates for embryos grouped by similar predictions. Flexible calibration (solid line) represents a smoothed estimate of observed success rates in relation to model predictions. The distributions of scores for positive and negative pregnancy outcomes are shown at the bottom of the graph

Figure 4 illustrates an example of a calibration curve based on simulated predictions from a hypothetical embryo evaluation model. As for all other evaluation metrics, the calibration curve should always be reported based on the independent test set. Predicted scores between 0 and 1 are shown on the *x*-axis, whereas corresponding success rates (pregnancy ratios) are shown on the *y*-axis. The triangles represent success rates for the evaluated embryos grouped by similar predictions (deciles). The red line represents a Loess smoothed estimate of observed success rates in relation to model predictions along with standard deviations [39]. The black, dotted line represents an ideal calibration. And finally, score distributions of embryos that result in positive and negative pregnancy outcomes are shown at the bottom.

In the specific example, we see that, on average, model predictions agree roughly with observed outcomes, as both the triangles and red line fluctuate around the ideal calibration. However, for model predictions below 0.5, the model is underestimating success rates, whereas for predictions above 0.5, the model is overestimating success rates. It thus seems that the model has overfitted on the calibration data (training or validation set). While different methods exist for measuring general lack of calibration, such as the Hosmer-Lemeshow goodness-of-fit statistic [40], these are all sensitive to both grouping methods (e.g., prediction deciles) and sample size, and provide no direction of miscalibration in terms of overestimation or underestimation. It is therefore generally recommended to present full calibration curves, as opposed to reporting a combined statistic [16, 38].

Calibration plots like the one in Fig. 4 can be used to assess the overall calibration of the model. They can, however, also be used to perform subgroup analyses by showing individual calibration plots for different age groups, clinics, fertilization methods (IVF/ICSI), etc. In this case, it may become evident that individual calibrations are needed for each group in order to match the observed outcomes.

Of the 13 studies presented in Table 1, only two considered the clinical agreement between model predictions and observed outcomes [22, 24], although in both cases without any mentions of the concept “calibration”. This agrees with a general trend for clinical prediction models that often neglect to report calibration results, but tend to focus entirely on discrimination [38].

In addition to calibration curves, clinical usefulness may also be addressed with decision curves [19]. As mentioned above, a single threshold may not generalize between different patients and clinical practices. Therefore, a decision curve such as net-benefit can show the expected benefit of a treatment (e.g., transferring an embryo) relative to no treatment as a function of the threshold value. The difficulty, however, lies in defining a harm-to-benefit ratio, incorporating all possible harms and benefits related to a treatment. In IVF, these could involve financial costs related to embryo cryopreservation, emotional costs related to transferring embryos that most likely do not result in pregnancies, or financial costs and extended time to pregnancy when deciding not to transfer at all, but instead starting a new cycle. As of now, however, none of these considerations seems to be quantified. That is, none of the studies listed in Table 1 reports patient-specific thresholds for deciding which embryos to transfer or cryopreserve and which embryos to deprioritize for transfer.

## Sample size

Traditional regression models often require, as a rule of thumb, at least 10 events per variable (EPV) when estimating sample sizes needed for model development [17]. A simulation study by [41] showed that machine learning methods such as neural networks often require at least 200 EPV to minimize overfitting. However, as argued in “?? ??,” modern AI models not only address overfitting issues by using “big data”. Training characteristics such as data augmentation, weight regularization, and potential pretraining on other datasets can greatly reduce overfitting and thus make the concept of EPV irrelevant. Instead, sample size considerations should be made when evaluating the generalization performance of a model. Here, the sample size of the test set determines what claims of performance can be made and with what certainty. For instance, for external validation of a prediction model, a sample size should be chosen that produces accurate and precise estimates of model performance [42].

**Fig. 5:**
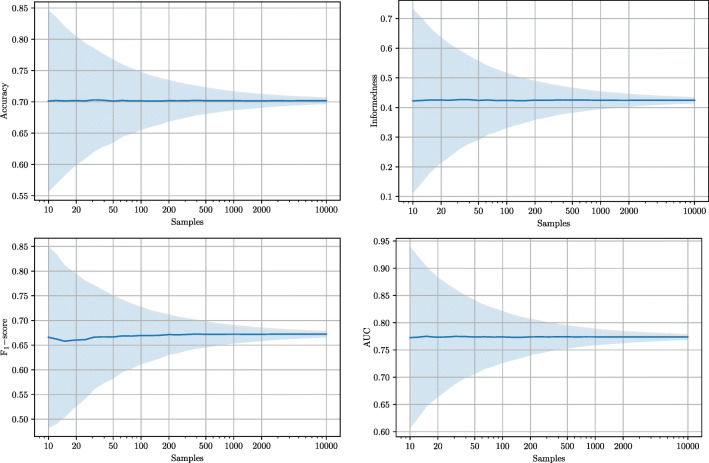
Influence of test set sample size (log scale) on standard deviations for different metrics. Solid lines denote mean values for each metric, whereas shaded regions illustrate the standard deviations. Potential performance improvements caused by increasing the sample size of the training set are not addressed in this analysis

Figure 5 illustrates this for four different metrics with simulated test sets of different sizes drawn from the hypothetical score distribution in Fig. 3a. Embryos are drawn randomly with an average prevalence of 40%. Accuracy, informedness, and F_1_-score are all calculated based on score thresholds of 0.5. For test sets with 10 embryos, all metrics have high standard deviations. This means that simply by role of chance, a test set with 10 embryos can be extremely easy or extremely difficult to distinguish for the same model. For test sets with 100 embryos, the variation is smaller with standard deviations around 1/3 of what they were at 10 samples. With 1000 embryos, all metrics have standard deviations of around 1/10 compared to the initial values at 10 samples. It is therefore important always to report confidence intervals for all performance measures, such that expected uncertainties are made explicit [16].

The above results illustrate that even if two AI models report their results on exactly the same population of embryos, sample size of the test set in itself greatly affects the certainty of a comparison, regardless of which metrics are used. Again, this underlines the importance of paired analyses, that is, evaluating different models on the exact same dataset.

## Bias in model comparisons

As shown in Table 1, embryo evaluation models are often compared to human embryologists. The purpose of these comparisons is generally to benchmark the AI model against the current manual selection/ranking procedure in the clinics. In this context, caution must be taken, as comparisons are most often made on retrospective data as opposed to actual prospective use under real clinical conditions [5]. This introduces a risk that the comparison may be biased by, e.g., optimism or reporting bias [17, 29, 43]. More so, results may be biased when the properties of the AI model was used to define the method of comparison. For instance, Chavez-Badiola et al. [13] compared their AI model against embryologists at predicting ploidy, even though embryologists are not trained for this task (illustrated by their performances that were similar to random predictions). Similarly, VerMilyea et al. [12] compared their AI model against embryologists based on binary (viable/non-viable) predictions. As embryologists were never trained or asked to provide such binary predictions, the authors instead inferred them from an existing scoring system used by embryologists, thereby introducing a risk of bias.

As indicated by the embryo population column in Table 1, the majority of evaluations are performed on a data subset of transferred embryos only. The decision to transfer these embryos has typically been performed by the embryologists themselves, possibly using the exact same (or a strongly correlated) procedure as the one used in the human vs. AI comparison. Unfortunately, this introduces a selection bias that is inevitable when comparing current and future practices in a retrospective cohort study.

**Fig. 6:**
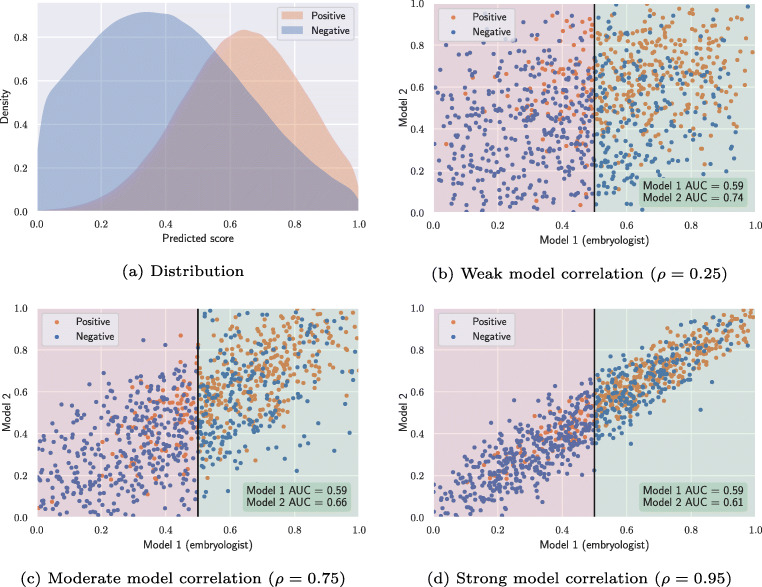
Influence of selection bias on model comparison

To illustrate the phenomenon, we have simulated how two similar models perform on a subset of embryos that was chosen by one of the models. Figure 6a depicts model score distributions of transferred embryos that result in positive and negative pregnancy outcomes. In this example, the two hypothetical models both assign scores with a mean of 0.65 and standard deviation of 0.2 for embryos with a positive outcome. Embryos with a negative outcome are sampled with a mean score of 0.35 and a standard deviation of 0.3. To simulate different correlations between the two models, all samples are drawn from bivariate truncated normal distributions with covariance matrices derived from Pearson correlation coefficients, *ρ*. We sample 1000 embryos of which 400 have positive outcomes and 600 have negative outcomes (prevalence = 0.4). As both models (model 1 and model 2) are sampled from the same score distributions, they also result in the same overall AUC of 0.76. That is, the two models are equally good at distinguishing positive and negative outcomes.

Figure 6b illustrates the scenario in which model 1 is used to select embryos for transfer in an actual clinical setting. Model 1 could thus represent embryo scores assigned by embryologists based on manual morphokinetic or morphological annotations. To simplify the analysis, we assume that all embryos with a score > 0.5 (green region) are transferred, while the rest are not used. When we evaluate model 1 on the transferred embryos only, the model now results in a lower AUC of 0.59. In many retrospective cohort studies, a newly developed AI model is compared against the traditional embryo selection procedure, retrospectively. This corresponds to evaluating model 2 on the subset of embryos that were chosen for transfer by model 1. On this subset, model 2 obtains an AUC of 0.74, which is considerably higher than the 0.59 obtained by model 1. Intuitively, this makes sense because model 1 has to discriminate all the transferred embryos based on scores between 0.5 and 1, while model 2 can still use the full range of scores between 0 and 1. In Fig. 6b, the difference in performance is large, because model 1 and model 2 are only weakly correlated. In practice, this means that the two models might not look at the same characteristics of an embryo, and thus they might assign substantially different scores to the same embryo. Figure 6c and d show similar examples, where the two models are moderately and strongly correlated. Here, we see that the performance differences narrow down, when the two models are more correlated. In the extreme scenarios, model 2 obtains an AUC of 0.76 (same as overall AUC) in case of no correlation *ρ* = 0, and 0.59 (same as model 1) in case of perfect correlation *ρ* = 1.

The simulation in Fig. 6 demonstrates selection bias in retrospective cohort studies. When a study compares its newly developed model (model 2) against a baseline model (model 1) that was used to select the test dataset (e.g., transferred embryos), the evaluation will be biased and falsely show better performance of the newly developed model. The amount of bias depends on two parameters: (1) the selection criteria of model 1, and (2) the correlation between the two models. The bias increases if the selection strictness of model 1 increases (e.g., a higher threshold than 0.5 in Fig. 6b–d). In practice, this means that for clinics with very strict selection strategies, AI models are more likely to appear better than humans without actually being so, simply due to the selection bias. The bias also increases if the correlation between the embryo scores of the two models decreases. While Fig. 6 only presents a comparison based on AUC values, Table 2 shows that the exact same selection bias exists for the summarized binary classification metrics accuracy, informedness and F_1_-score (when choosing optimal thresholds for each metric).

**Table 2:** Influence of selection bias on model comparison with different performance metrics

		Accuracy			Informedness			F1-score			AUC	
		Model 1	Model 2		Model 1	Model 2		Model 1	Model 2		Model 1	Model 2
Overall		0.70	0.70		0.41	0.41		0.68	0.68		0.76	0.76
*ρ* = 0.25		0.60	0.71		0.13	0.37		0.74	0.79		0.59	0.74
*ρ* = 0.75		0.60	0.66		0.14	0.25		0.74	0.76		0.59	0.66
*ρ* = 0.95		0.61	0.63		0.14	0.15		0.75	0.75		0.59	0.61

All performance measures are obtained from the simulations in Fig. 6

In the above simulations, a global threshold of 0.5 was used to decide whether embryos should be transferred or discarded. This served as a simple criteria, which was easy to visualize. In reality, such a global threshold is never used. However, even in realistic cases with less strict transfer-policies, the selection bias still exists. And even when the baseline model (model 1) of a study is not exactly the same as the model or procedure that was used to select the test dataset, the selection bias remains in place if the two procedures are correlated. For instance, if the decision to transfer certain embryos was based on blastocyst grading and the baseline model (model 1) used both blastocyst grading and morphokinetics, they are still expected to correlate, and thus still introduce a selection bias in the comparison.

Many of the papers listed in Table 1 present comparisons of their AI models against embryologists on retrospective data without any mentions of biased performance considerations [12–15, 20, 22, 24, 26]. Some of these even claim statistical significant superiority over embryologists [12, 13, 20]. However, in order to eliminate selection bias and thus provide a fair comparison, randomized controlled trials are needed.

## Discussion

Modern AI techniques for embryo evaluation have the potential to both automate and improve current manual and subjective selection performance. This will result in an improvement of the clinical workflow, reduced time spent on manual evaluations, and possibly even a reduction in time to pregnancy. In recent years, several studies have reported promising results using artificial intelligence (AI) to automatically analyze embryo images or videos. The objective of these methods can be to (1) rank embryos according to their potential to implant, and/or (2) predict the actual probability of pregnancy for each embryo. How to evaluate the performance of these objectives, however, is currently inconsistent across studies, and recommendations and best practices on the subject have not yet been agreed upon.

In this paper, we have shown that it is not possible to define a set of “universal” requirements and recommendations for how to split datasets into training, validation and test sets. Neither is there a universal truth for whether to balance datasets in order to ensure an equal numbers of positive and negative examples. That is, unbalanced datasets can cause problems during training and seemingly provide overoptimistic performance measures of prevalence-dependent metrics during evaluation. At the same time, balanced datasets do not represent actual clinical practices, as current success rates of IVF are often below 50%.

We have provided a list of the most common evaluation metrics and related all of them to their dependency on prevalence (data balance). Relating the metrics to the two objectives of (1) ranking and (2) prediction, we recommend the area under the receiver operating characteristic (ROC AUC) for reporting overall ranking performance across treatments. Furthermore, when sufficient outcomes are available within a treatment (e.g., for preimplantation genetic testing), normalized discounted cumulative gain (nDCG) can be used to report ranking performance on treatment level. To evaluate the performance of probabilistic prediction models, we recommend calibration curves, possibly accompanied by decision curves to document the clinical relevance of providing probability estimates. Further reporting, such as showing the ROC curves and not just reporting AUC values, may also help scientists and clinicians assess discrimination performance at various thresholds. However, providing a single confusion matrix or single values for binary metrics such as accuracy, sensitivity, or specificity, can be misleading, as no single model threshold is likely to generalize across all patients and clinical practices.

According to several recent studies listed in Table 1, current AI models already surpass human performance [12, 13, 20] and are hypothesized to provide improvements of clinical relevance as well [12]. Additionally, some studies claim superiority over others, by comparing reported measures across datasets, embryo populations, and measured outcomes.

In this paper, however, we have illustrated how AI model evaluations on different embryo populations or measured outcomes cannot be compared in a meaningful way. That is, different data distributions, patient populations, or observed success rates directly affect maximum obtainable performance measures and thus are incomparable. Therefore, paired analyses on the same datasets seem to be the only appropriate and valid comparisons available. Ideally, a large multicentric dataset hosted by an independent party could be used to evaluate and compare AI models in a double-blinded fashion. In this way, embryo image data would be publicly available, whereas outcome labels would be hidden and managed centrally just like in the ImageNet challenge [32]. Using this approach, different AI models could be compared on various subpopulations for their performances on both ranking and probability prediction. However, we have also shown that even in the case of a paired analysis, selection bias is inevitable when comparing current and future practices on transferred embryos in a retrospective cohort study. In practice, this means that superiority claims, even in case of statistical significance, should always be interpreted with caution. Along with many other concerns such as model generalization ability, clinical relevance, and potential model bias, this finding highlights the importance of prospective trials. To truly validate the performance of an AI model in a clinical context and to reveal any improvements over current practices, randomized controlled trials is the only valid evaluation.
